# Editorial: Timetrees: Incorporating fossils and molecules

**DOI:** 10.3389/fgene.2022.937763

**Published:** 2022-06-30

**Authors:** Michel Laurin, Gilles Didier, Rachel C. M. Warnock

**Affiliations:** ^1^ UMR7207 Centre de recherche en paléontologie - Paris (CR2P), Paris, France; ^2^ IMAG, Univ Montpellier, CNRS, Montpellier, France; ^3^ ETH Zürich, Zurich, Switzerland

**Keywords:** molecular dating, tip-dating, fossil record, birth-and-death models, geological age

Calibrating phylogenies to time is central to addressing many questions in evolutionary biology and macroevolution, such as the timing and dynamics of evolutionary radiations (e.g., Brocklehurst, 2017; Ascarrunz et al., 2019; Didier and Laurin, 2020) and of mass extinction events and their possible environmental causes (e.g., Allen et al., 2019; Didier and Laurin, 2021). The fossil record once provided our only source for establishing a timeline for evolution (Romer, 1966), but the incompleteness of this record and its non-uniformity in space and time limit the precision of divergence time estimates (Laurin, 2012; Heath et al., 2014; Warnock et al., 2017; Didier and Laurin, 2020). Molecular dating, which combines evidence from the geological and molecular records, can generate a much more complete and precise timeline of events (e.g., Sauquet, 2013; Magallón, 2020). This Research Topic focuses on recent advances in methodology, outstanding challenges, and the application of molecular and paleontological dating methods to empirical case studies across the Tree of Life.

Marshall reviews paleontological approaches to estimate divergence times, pointing out the many difficulties arising from this task. Though minimum ages are quite straightforward to infer from the fossil record, maximum age constraints are not so easy to establish. A first point to keep in mind is that the fossil record informs only about the first (fossilized) apomorphy and not the actual divergence time. Other major issues arise from the fact that the fossil recovery rate is not homogeneous and varies substantially over time and space. Marshall discusses various approaches to deal with these difficulties and shows some examples of paleontological dating.

Matschiner performs simulations in order to assess the influence of selective sampling of fossils or extant species on the accuracy of divergence times inferred under the Fossilized Birth-Death (FBD) model. He observes that non-uniform sampling of fossils or extant taxa leads to biased estimates of node ages obtained from the FBD model, notably in the case where the fossil record is reduced to the oldest fossil of each branch. Another node dating approach, called CladeAge (Matschiner et al., 2016), shows better behavior in the presence of selective sampling of taxa in simulated data (but see Zhang et al., 2016).

Barido-Sottani et al. use simulations to examine the impact of fossil age uncertainty on trees recovered using the FBD model for fully extinct clades. They show that fixing fossil ages to a point age within the known range of stratigraphic age uncertainty produces incorrect estimates of both topology and divergence times. They also illustrate the impact of different approaches to handling fossil age uncertainty on parameter estimates among a group of Paleozoic crinoids. They further demonstrate that best solution is to explicitly model fossil age uncertainty.

Guindon provides a general presentation of molecular dating methods based on various assumptions (namely, strict, not-so-strict, uncorrelated and autocorrelated, relaxed clock models). He next reviews several approaches to calibrate clock models, mainly based on fossil records. After a brief presentation of how to process fossil for use in this context, he presents and discusses various model-based calibration methods, pointing out some issues in using the FBD model.

Powell et al. assess the advantages and drawbacks of secondary calibrations (which are molecular estimates of divergence times obtained in previous studies) compared to more distant primary (i.e., paleontological or geological) calibrations. This is timely because for many taxa with a poor fossil record (typically those containing organisms lacking a mineralized skeleton), calibration can be performed only through one of these alternatives. They find that distant primary calibrations provide better precision, but note that secondary calibrations remain useful.

Lozano-Fernandez et al. explore hypotheses about the geological context surrounding the colonization of land by arachnids. They generate a large dated tree of arachnids based genome-scale sequence data and a suite of rigorously assessed node calibrations. The origin of arachnids is dated to the Cambrian or Early Ordovician, indicating that terrestrialization occurred within this interval. This is followed by a rapid radiation of the group, coincident with elevated rates of molecular evolution. The authors suggest that the outstanding discrepancy between molecular estimates for the origin of crown group arachnids and the first appearance of body fossils belonging to this group can be attributed to incompleteness of the early terrestrial record.

Marjanović highlights problems associated with obtaining reliable time calibrations for node dating, caused by rapid progress in paleontology, thus rendering the few compilations (e.g., Benton et al., 2015) of such calibration constraints soon out of date, as more fossils are discovered or the information is updated. But worse, some molecular studies copy such constraints from previous molecular studies that had not necessarily used the most recent paleontological literature. These problems are illustrated through a detailed analysis of the 30 calibrations used to produce the largest available vertebrate timetree (Irisarri et al., 2017).

Pardo et al. assess the problems in obtaining reliable ages for three main crown-clades of limbed vertebrates (Tetrapoda, Lissamphibia and Amniota) to calibrate molecular clocks. They show that whereas much emphasis has been placed recently on documenting the age of fossils and providing synapomorphies that prove that they belong to a given clade (Parham et al., 2012), the main problem with deep tetrapod nodes is that the phylogeny is controversial and that various alternatives imply different ages for these clades.

Springer et al. review evolutionary models for the diversification of placental mammals, which differ from each other in the proposed timing of the evolutionary radiation of crown-placentals relative to the K/Pg boundary. At one extreme, this whole radiation may have started soon after the K/Pg boundary and proceeded very quickly, whereas at the other end of the spectrum, this radiation started around the mid-Cretaceous. Many problems (e.g., establishing homology of molecular sequences, taxonomic affinities of fossils and validity of the morphological clock) affect some or all of the three main dating methods (node-, tip-, and fossilized birth-death dating).

Celik and Phillips examine incongruence in the phylogeny of mammals based on different anatomical regions. This incongruence is attributed to convergent and correlated character evolution within ecologically similar but phylogenetically distinct groups. The authors develop a metric (the maximum parsimony disadvantage score) that allows us to identify homoplasy within anatomical partitions. They find that within mammals, cheek teeth and shoulder girdle characters have high potential to mislead phylogenetic inference due to non-phylogenetic covariance within these regions. These results have implications for assessing the placement of mammal fossils and consequently their inclusion in molecular dating studies.

Finally, Paterson et al. re-examine the monophyly of pinnipeds, which were widely believed to be diphyletic from the 1960s to the 1980s, and to assess parallel evolution within the group. Their Bayesian (as well as parsimony) analyses confirm pinniped monophyly but also demonstrate a surprising amount of parallel evolution in characters that had previously been interpreted as pinniped synapomorphies. These include dental and limb bone characters relating to homodonty and aquatic locomotion, respectively. New tip-dating analyses date the divergence between pinnipeds and musteloids to about 45 Ma.

Together, these studies illustrate the utility of timetrees in addressing fundamental questions about evolution, as well as underscoring the need to apply a rigorous approach to select calibrations, models and prior parameters.
